# The Future Toolbox for Managing Ketosis in Dairy Cow Herds: A European Key Opinion Leader Consensus

**DOI:** 10.3390/vetsci13040344

**Published:** 2026-04-01

**Authors:** Celien Kemel, Angelique C. M. Rijpert-Duvivier, Nina Strus, Florian Guigui, Frédéric Vangroenweghe

**Affiliations:** 1 Department of Internal Medicine, Reproduction, and Population Medicine, Faculty of Veterinary Medicine, Ghent University, Salisburylaan 133, 9820 Merelbeke, Belgium; celien.kemel@ugent.be; 2 Elanco Animal Health Benelux, BU Ruminants & Swine, Generaal Lemanstraat 55/3, 2018 Antwerp, Belgium; 3 Elanco Animal Health Central & Eastern Europe d.o.o., BU Ruminants & Swine, Dunajska Cesta 167, 1000 Ljubljana, Slovenia; 4 Elanco Animal Health France, BU Ruminants & Swine, Place de Belgique 10, Paris-La-Défense, 92250 La Garenne Colombes, France; florian.guigui@elancoah.com

**Keywords:** dairy cows, data-driven decision-making, hyperketonemia, ketosis, proactive management, transition health, veterinarian–farmer collaboration

## Abstract

Ketosis is a major metabolic disorder that significantly impacts dairy cow health, welfare, and farm profitability, posing challenges to both farmers and veterinarians. This opinion paper, derived from expert panel discussions and a review of the scientific literature, provides a comprehensive, proactive approach to modern ketosis management. It addresses the critical need for increased farmer awareness, emphasizing the veterinarians’ involvement as consultants and data interpreters and equipping them with essential skills in data analysis, communication, and farmer education. This paper also details a practical toolbox of diagnostic, therapeutic, management, and preventive strategies, including precision technologies and welfare-enhancing practices, to optimize metabolic health, enhance productivity, and ensure the long-term sustainability of dairy farming. This expert consensus translates scientific knowledge into practical on-farm actions, empowering farmers with risk-based insights and equipping veterinarians with tools and strategies for success. Ultimately, the consensus of our opinion paper reflects an industry-wide transition toward absolute transparency in diagnostic reporting, based on reliable data that creates an indispensable foundation for evidence-based ketosis management.

## Simple Summary

Ketosis is a major metabolic disorder that significantly impacts dairy cow health, welfare, and farm profitability, posing challenges to both farmers and veterinarians. This white paper provides a comprehensive, proactive approach to modern ketosis management. It addresses the critical need for cooperation between farmers and veterinarians to deal with the disease. The paper also details a practical toolbox for the approach of ketosis management. This expert consensus translates scientific knowledge into practical on-farm actions, empowering farmers with risk-based insights and equipping veterinarians with the tools and strategies for success.

## 1. Introduction

Most dairy cows pass through a period of negative energy balance (NEB) during the transition from late gestation to the onset of lactation. Poor adaptation during this period is expressed by elevated concentrations of non-esterified fatty acids (NEFAs) in the close-up period and/or high concentrations of ketone bodies (beta-hydroxybutyrate (BHB)) within the first 3 weeks post-partum, known as hyperketonemia. The term hyperketonemia refers to any increase in the blood concentration of ketone bodies (BHB) that is greater than those considered physiologically normal, whereas in the literature, different thresholds are discussed, ranging from 1.0 to 1.4 mmol/L, which are measured between 3 and 16 days in milk (DIM) [1,2,3,4]. The level of ketone bodies in milk (BHB) and urine (acetoacetate) measured in the first 3–16 DIM shows a significant positive correlation with the blood BHB concentrations (with reported correlation coefficients ranging from 0.51 to 0.84), supporting their use as reliable on-farm diagnostic proxies [5]. However, the measurement of blood BHB concentration is considered the gold standard for the diagnosis of hyperketonemia in dairy cows. Milk and urine testing can be useful and are easier alternatives for on-farm monitoring, but their sensitivity and specificity are somewhat lower compared to blood BHB measurements [6]. Hyperketonemia does not describe a clinical presentation; it is not a disease in a strict sense but rather an elevated concentration of a metabolic marker that can be tested. This definition differs from clinical ketosis, which is often used to indicate a clinical postpartum condition with blood ketone levels higher than 2 or 3 mmol/L, resulting in clinical symptoms such as decreased appetite, weight loss, and decreased milk production, with nervous signs in severely hyperketonemic cows affecting only a low percentage of the animals. However, subclinical ketosis (SCK) indicates animals with a blood BHB concentration exceeding the “normal” threshold, without obvious clinical signs, but affecting a considerably higher percentage of the animals [3,7]. To maintain terminological consistency throughout this publication, the metabolic disorder characterized by the blood BHB concentrations exceeding 1.2 mmol/L within the first 21 days of lactation or the test results in other media that exceed the thresholds that were validated against a BHB concentration of 1.2 mmol/L in blood will be uniformly referred to as ketosis.

A certain level of ketone bodies in the bloodstream at the onset of lactation is a marker of the physiological adaptation of a dairy cow in the early postpartum period to her negative energy balance, where they can serve as an alternative fuel source. However, when the ketone bodies exceed certain limits, this condition can be harmful, and it is associated with clinical symptoms and undesirable outcomes, with many studies showing the negative impacts of these elevated ketone levels on cow health, fertility, and milk production. However, the recent literature presents a more complex picture, showing that some highly productive cows have high ketone body levels without clear detrimental effects, while others seem to suffer negative consequences. Researchers have hypothesized that high levels of ketone bodies may occur as a consequence of underlying physiological or inflammatory processes or immune activation, rather than being the primary cause of negative outcomes. A further investigation into this relationship is necessary, particularly as ketosis in the first week of lactation often indicates underlying physiological or inflammatory issues, whereas cases occurring from the second week onwards are typically associated with the metabolic demands of high milk production [8,9,10]. Regardless of the timing, ketosis remains highly prevalent across European dairy herds [11].

This publication is based on a structured expert panel discussion that was held in May 2025, involving 13 recognized subject matter experts from nine key EU dairy markets (Belgium, the Czech Republic, France, Germany, Hungary, Poland, Portugal, Spain, and the Netherlands). Using the Nominal Group Technique (NGT), this structured, multi-step methodology ensured an objective synthesis of expert opinion through independent idea generation, facilitated group discussion to clarify diverse viewpoints, and a final ranking phase to resolve disagreements. The participants were selected based on their demonstrated expertise in ruminant metabolic health, representing a balance between academic research (including the faculty members from the Universities of Ghent, Berlin, and Krakow) and specialized clinical practice that is focused on data-driven herd health management and proactive farm advisory services. This process allowed the panel to establish a unified ‘Future Toolbox’ and provide recommendations for enhanced ketosis management through farmer–veterinarian collaboration. The expert panel’s discussions were informed by a comprehensive, targeted review of the scientific literature, focusing on European ketosis prevalence, diagnostic accuracy, and economic impacts. While not a formal systematic review, this evidence-based approach ensured that the resulting consensus and the ‘Future Toolbox’ were supported by high-impact, peer-reviewed data [1,2,3,4,5,6,7,8,9,10,11,12,13,14,15,16,17,18,19,20,21,22,23,24,25,26,27,28,29,30,31,32,33,34,35,36,37,38,39,40,41,42,43,44,45,46,47,48,49]. It addresses: (1) the awareness of ketosis in dairy farming, (2) the veterinarian’s role in ketosis control, and (3) the essential tools for ketosis management. This paper aims to guide veterinarians and farmers on effective ketosis management, offering insights into essential skills, tools, and strategies. It also informs relevant stakeholders by highlighting critical areas for intervention and offering essential market intelligence for the development of novel solutions. Ultimately, this work converts subject matter expertise into evidence-based recommendations for improving metabolic status, maximizing dairy performance, and ensuring farm sustainability.

## 2. Ketosis Awareness in Dairy Farming

The expert panel stressed the need for increased ketosis awareness due to the disparity between its high prevalence [11] and limited farmer recognition. This stems from ketosis’s largely subclinical nature and the practical difficulties of implementing effective monitoring using existing diagnostics. To enhance awareness, the panel recommended: (1) standardizing terminology, (2) elevating farmers’ understanding of ketosis’s impact, (3) farm-specific databases, and (4) empowering farmers with action lists based on individual cow risk assessments.

### 2.1. Standardized Terminology for Enhanced Communication

Clear communication requires a standardized lexicon, especially given the subclinical nature of ketosis and the many different definitions found in the literature. While physiological considerations are relevant, the panel notes that these may be overly complex and less effective as a primary call to action.

Panel recommendations:

• Vets: They are to agree with farmers on simple ketosis definitions (e.g., clinical vs. subclinical) based on the chosen testing method and use these consistently in reports, software, and discussions.
• Farmers: They are to record all the health events and test results using these same agreed definitions and thresholds so that ketosis trends can be reliably monitored over time.

### 2.2. Elevating Farmer Understanding of Ketosis’s Impact and Subclinical Nature

The panel emphasizes a key aspect of increasing ketosis awareness, which is educating farmers about its significant impact. While clinical signs are observed in 2–15% of cows [7], the global prevalence of subclinical ketosis is substantially higher (22.7%) [10], often underestimated by farmers (expert panel). This discrepancy arises because over 80% of cows with elevated BHB levels show no visible signs [2,12], necessitating the testing of ketone body levels in blood, milk, or urine. Furthermore, a solid understanding of the chosen method, including the sensitivity and specificity of the diagnostic test, the medium tested, the selected thresholds, the cohort of animals, and the time frame of testing, is essential for interpreting the results and identifying the animals that need support. Therefore, comprehensive farmer education is fundamental and should encompass the impact of elevated ketone levels on herd performance (reduced milk production by 1.2 kg/day in early lactation, reproductive issues, three-fold increase in the risk of secondary disorders, such as displaced abomasum [2,12,13,14], the associated economic losses) (estimated €18–812/cow, as stated in various studies published before 2018) [15] and help them understand the negative implications for animal welfare and sustainability [16].

Panel recommendations:

• Vets: They should make ketosis a standard topic at every herd health visit, use farm data to show that most cases are subclinical, and explain the main economic and welfare impacts.
• Farmers: They should ensure that all staff working with transition cows are trained to recognize risk periods, know that testing is needed to detect most cases, and can correctly use and interpret simple ketone tests.

### 2.3. Farm-Specific Data for Enhanced Decision-Making

The expert panel emphasized the necessity of farm-specific data for effective decision-making. The personalized datasets are deemed more impactful than the generalized prevalence data, although the latter can provide valuable benchmarking comparisons. The experts identified two primary approaches to developing these databases:

First, to utilize the existing farm data, such as dairy herd improvement (DHI) records, which requires the vets to analyze and present this information in a clear, concise, and actionable format for farmers. Second, implementing new monitoring technologies, such as incorporating milk fatty acid profiles that are determined by Fourier transform infrared (FTIR) spectroscopy into DHI reports, as this offers opportunities for more targeted and comprehensive data collection. This development is important because the use of other technologies, for example, HPLC methods, is costly and time-consuming [17]. The use of FTIR-based predictions, although less accurate than the golden standard, allows for much larger-scale studies of the associations between fat composition and health [18,19].

The standardized protocols are critical for data comparability, cost-effectiveness, and maximizing return on investment (ROI). A consistent on-farm data collection establishes monitoring routines, which facilitate proactive management.

Panel recommendations:

• Vets: For each herd, they should set up a basic ketosis monitoring protocol that combines existing farm data (milk recording, disease, and treatment records) with targeted ketone tests in early lactation, and they should present key indicators in a simple visual format.
• Farmers: They should maintain complete transition cow records and review the monitoring results with the vet at agreed intervals to decide whether management or feeding practices need adjustment.

### 2.4. Empowering Farmers in Recognizing At-Risk Animals and On-Farm Risk Factors

The expert panel points out that a highly effective strategy for raising ketosis awareness involves empowering farmers with action lists based on individual cow risk assessments. This helps to routinely implement targeted preventive measures. The expert panel underscored the importance of addressing the risk factors at both the herd and individual cow levels. Frequent pen changes, the introduction of new animals, overcrowding, and an imbalanced transition cow ration are important risk factors for ketosis at the herd level [20]. At the cow level, third parity and older cows, cows with body condition scores higher than 3.5, and animals with a history of transition diseases are at higher risk [21]. The veterinarian’s role is essential in prioritizing these risk factors within the specific farm context, ensuring that interventions are targeted. Other key roles concerning ketosis awareness on farms can be identified, such as the nutritionists who are responsible for formulating the feeding rations. A close cooperation between professionals is highly recommended to increase awareness.

Panel recommendations:

• Vets: They should create short herd- and cow-level ketosis action lists with the farmer (and nutritionist) and review/update them regularly based on results.
• Farmers: They should apply the agreed herd actions (e.g., limit regrouping, avoid overcrowded transition pens, feed suitable transition rations) and keep an up-to-date list of high-risk cows for closer observation, prevention, and targeted ketone testing.

## 3. The Veterinarian’s Critical Role in Ketosis Control

Given the need for an increased ketosis awareness as identified by the panel, the discussion now turns to the veterinarian’s essential role in effective ketosis control. The expert panel emphasizes that the implementation of comprehensive ketosis control programs requires a set of core skills for veterinarians that are not unique to ketosis (e.g., communication, advisory, and data skills), but which must be explicitly applied to the ketosis monitoring, decision-making, and preventive management on farms. The modern dairy veterinary practice, particularly in high-production herds, is evolving from a focus on individual animal treatment (e.g., lameness and mastitis) [22] towards herd-level monitoring and preventive care. While specialization remains important [23], veterinarians are increasingly becoming trusted advisors on herd management and improving the overall health and welfare of the cows [22,24,25]. The expert panel focused on two key areas for the dairy veterinarians’ involvement in ketosis control:

1. Veterinarians transitioning to proactive advisory and welfare stewardship.
2. Essential skills for future dairy veterinarian success.

### 3.1. Transitioning to Proactive Advisory and Welfare Stewardship

**Figure 1:**
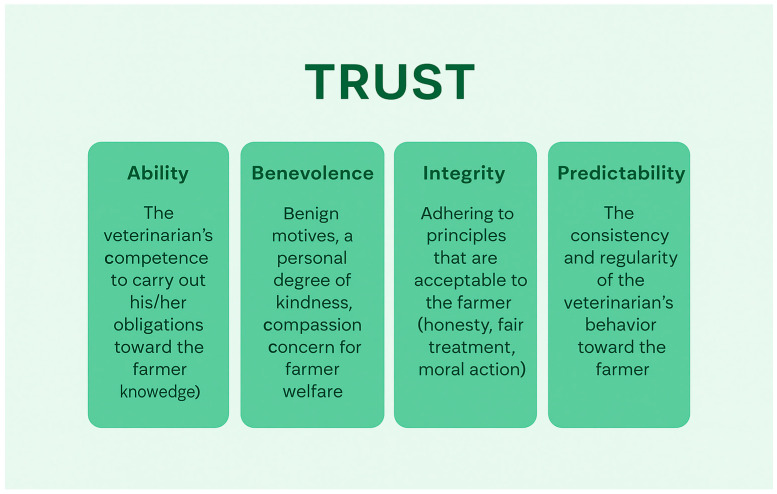
The four virtues needed for the assessment of veterinarian trustworthiness by farmers (adapted from Bard et al. [24]).

The expert panel unanimously agreed that dairy veterinarians must shift their focus from a reactive “firefighter” role to a proactive, holistic farm advisor, integrating various aspects of dairy management. This aligns with the increasing global emphasis on veterinarians’ contributions to One Health [23] and the need for independent advice, subclinical disease risk management, and team-based problem-solving at the farm level [16]. Similarly, a shift in farmer mentality from individual cow treatment to herd-level prevention is fundamental. Building trust, considering farmer priorities, and fostering a positive behavioral change are the keys to effective veterinary advice [24] (Figure 1). Recognizing the value of this partnership, appropriate compensation for the veterinarian’s time, knowledge, and services is essential for its long-term viability in general and for it to work effectively to improve the metabolic health of the dairy cows.

The expert panel maps out that veterinarians are important to advocate for animal welfare on dairy farms. This aligns with the results of a Canadian study where a survey was executed amongst veterinarians and veterinary students. The respondents indicated that health, welfare, and being an advocate for the animal are the most important facets for a veterinarian to consider [22]. Furthermore, veterinarians recognized their role in food safety and consumer concerns. The veterinarians acknowledged their unique professional standing; however, a common pitfall or occupational hazard is the neglect of their personal well-being.

### 3.2. Essential Skills for Future Dairy Veterinarian Success

The second key area of the expert panel’s discussion focuses on the skills that dairy veterinarians need for effective farmer partnerships.

**Data handling:** These skills center around data monitoring, analysis, and management to make decisions and recommendations for improved ketosis management. The expert panel’s opinion is supported by recent publications; as data-driven veterinary intelligence systems become increasingly important for predicting the status of animal health and enabling the preventive measures [26], veterinarians must develop strong data interpretation skills. This is necessary because, while over 50% of farmers utilize or plan to utilize technology for recording farm animal activity (e.g., rumination and movement) [25], many struggle with data interpretation [27]. Veterinarians must bridge this gap, leveraging their expertise in information and communication technologies to effectively incorporate farm data into daily farm practice [27]. For example, with appropriate data, veterinarians can demonstrate the cost/benefit relationship of ketosis management by calculating the economic impact of SCK, which was estimated at €257 per cow in a study published in 2015 [28]. When farmers understand their farm’s data, they can be trained to recognize key ketosis risk factors, such as those related to parity (third parity and older cows), body condition score (>3.5), and previous transition cow diseases [7,29,30,31,32] and implement appropriate preventive measures such as targeted nutritional supplementation, targeted preventative treatment, dry-cow ration optimization, and stocking density adjustments. This data-driven approach, combined with clear communication and farmer education, allows veterinarians to implement tailored solutions, including disease prediction software (using AI), targeted SCK treatment, and preventive protocols for at-risk cows.

**Educational skills:** Furthermore, the expert panel emphasized the need for effective farmer education. To achieve this, veterinarians must translate complex scientific knowledge into easily digestible information, using pedagogic skills and marketing principles to craft compelling narratives, create impactful educational materials, and facilitate clear communication that motivates farmers to action. Motivational interviewing is proposed in the literature to encourage a behavior change in farmers and herd managers [33,34].

**Figure 2:**
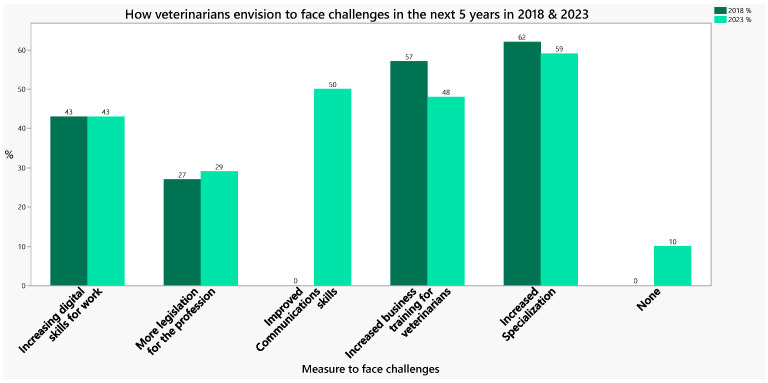
The potential ways that veterinarians will meet challenges in the next 5 years (adapted from FVE Survey, 2023) [23].

**Communication skills:** By combining the aforementioned skills with effective communication strategies, veterinarians can empower farmers to understand and address ketosis. The panel considers these tools essential skills in the veterinarian’s toolbox for ketosis. These capabilities help to embed ketosis management as an explicit, structured component of the herd health and welfare approach. Like our expert panel’s view, the 2023 FVE Survey shows that European veterinarians believe there is a strong demand for improved communication, business, and digital skills, along with further specialization [24] (Figure 2).

## 4. Essential Tools for Modern Ketosis Management in Dairy Cows: Equipping Veterinarians and Farmers for Success Expert Panel Recommendations: A Proactive Approach

The expert panel’s recommendations focused on proactive ketosis management, aligning with the current trends in animal welfare science [24]. This proactive approach aims to minimize the negative impact of ketosis and contribute to improved health and productivity.

Table 1 and Table 2 provide comprehensive overviews of tools and strategies for effective ketosis management in dairy cows for veterinarians and farmers, respectively. It integrates recommendations from the expert panel, supporting evidence from the scientific literature, and highlights the preventive potential of each element. While the literature emphasizes the importance of early detection [10,35,36,37,38,39] and targeted interventions [40,41,42], the expert panel concluded that the practical implementation of these tools often fails due to a lack of clear communication protocols between the veterinarian and the farmer. Consequently, the following toolbox focuses on bridging this gap through structured, collaborative actions. In both toolboxes, one could argue that most tools need the involvement of the veterinarian and the farmer. When the data derived from on-farm monitoring protocols or sensor output necessitates complex analysis, a veterinary consultation is essential to provide a clinical interpretation and for diagnostic validation. Furthermore, while the veterinarian is responsible for the clinical prescription and protocol, the farmer typically executes the administration. Consequently, these therapeutic interventions serve as practical management tools for the farmer, reinforcing the collaborative nature of ketosis control. In conclusion, cooperation in ketosis management between these professionals is key.

## 5. Conclusions

This opinion paper, reflecting the consensus of a European expert panel, underscores the critical need for a proactive and comprehensive approach to ketosis management in dairy cows. The panel highlighted the importance of increasing farmer awareness of ketosis, particularly its subclinical form, through standardized terminology, targeted education emphasizing economic and welfare implications, and data-driven decision-making using farm-specific information. Addressing these implications is vital, as the economic burden of ketosis can reach up to €812 per cow due to reduced milk yield, impaired reproductive performance, and a higher incidence of secondary diseases such as displaced abomasum, which can impact welfare. Furthermore, the evolving role of the veterinarian as a trusted advisor and data interpreter was emphasized, demonstrating the need for enhanced skills in communication, data analysis, and farmer education, potentially incorporating techniques like motivational interviewing. Crucially, the panel identified a clear need for effective, evidence-based preventive solutions, spanning diagnostic tools, therapeutic interventions, and management strategies, to mitigate the negative impacts of ketosis and optimize cow health and productivity. Future research should focus on refining diagnostic capabilities, developing targeted preventive treatments, and optimizing management protocols that integrate precision technologies and welfare-enhancing practices and will further bridge the gap between scientific knowledge and practical on-farm application, empowering both farmers and veterinarians. In conclusion, the future toolbox for managing ketosis in dairy herds includes a structured use of existing tools that have been implemented in proactive herd health approaches. Furthermore, the future toolbox for ketosis includes the veterinarian being competent in data interpretation, sensor technologies, communication, and farmer education, so they can turn complex outputs into clear, farm-specific ketosis plans that link diagnosis, monitoring, treatment, and prevention. Veterinarians and universities should make efforts to enhance the structured use of the tools available and improve the necessary capabilities. To prevent ketosis, the toolbox for individual at-risk cows still needs to be expanded with effective, evidence-based interventions. Strengthening this toolbox will help veterinarians safeguard animal health, welfare, and food safety, while enabling farmers to understand their own ketosis situation, implement agreed protocols, and improve animal health, profitability, and the sustainability of their herd. A practical, summarized recommendation for veterinarians and farmers based on this opinion paper is available in the Supplementary Materials.

## Figures and Tables

**Table 1:** The comprehensive ketosis management toolbox primarily for the veterinarian, integrating expert recommendations and preventive strategies.

Tool Type	Specific Tool/Approach	Description	Expert Panel Emphasis/Literature Support	Preventive Intervention Potential
**Diagnostic**	Blood, urine, or milk ketone measuring.	Rapid on-farm assessment of blood or milk BHBA levels or urine acetoacetate levels. Milk BHB offers high diagnostic utility with reported sensitivity and specificity, often exceeding 80% and 90%, respectively, when compared to blood BHB. Testing needs to be standardized per herd, considering the diurnal rhythm of the BHB concentration in the different body fluids and preferably in the first week post-calving.	[10,35].	Limited direct preventive potential, primarily for diagnosis.
	Indicators of inflammation (haptoglobin concentration in blood post calving).	A recommendation to investigate further when measuring acute phase proteins can help to indicate future disease and performance issues at the cow level and to decide on treatment.	[36].	Limited direct preventive potential, primarily for diagnosis and understanding the underlying metabolic status.
**Monitoring**	Milk, blood, or urine ketone tests.	Herd-level screening for subclinical ketosis can be incorporated into routine milk recording or even into in-line milk recording. Routine prevalence testing via blood or urine in all cows or in batches of freshly calved cows can be used as a monitoring instrument.	In-line milk tests, cow-side biomarker assessments, automated monitoring systems, and real-time data analysis advocate for early detection and estimation of prevalence (expert panel consensus) and [22].	Supports prevention through monitoring.
	Metabolic profiling (e.g., fatty acids, non-esterified fatty acids (NEFA’s), IGF-1, acute phase proteins).	A comprehensive assessment of the metabolic state requires specialized laboratory analysis.	[37,38].	Indirectly supports prevention through improved understanding of metabolic pathways, potentially leading to targeted nutritional or preventive interventions.
**Monitoring**	Monitoring of fresh cow health.	Close observation by clinical examination in the first weeks of lactation for early detection of ketosis.	Early detection is critical (expert panel consensus) and [39].	Supports early intervention to minimize the impact of ketosis.
	Multimodal data integration: integration of data from various farm systems.	Enabling comprehensive monitoring and analysis of herd-level ketosis risk factors.	Identification at risk herds (expert panel consensus).	Supports preventive management interventions at the herd level.
**Treatment/ ** **prescription**	Propylene glycol.	Targeted oral propylene glycol treatment in subclinical cases has been shown to reduce the risk of displaced abomasum by 40% and increase milk yield by 0.5 kg/day during the first 30 days of lactation compared to untreated controls.	[40,41,42].	Mainly used as treatment of ketosis for 3–5 days.
	Glucose precursors (e.g., calcium propionate).	Oral or IV administration stimulates gluconeogenesis.	[43].	Short-term treatment, not a preventive measure.
	B vitamins (especially B12 or combined with butaphosphan).	Injections to support metabolic function.	[41,42,44].	Supportive therapy may contribute to prevention if used strategically alongside other measures.
**Management/** **Prevention**	Body condition scoring (BCS).	Regular monitoring of BCS throughout lactation helps identify cows at risk and guide nutritional adjustments.	Expert panel consensus.	Important for identifying at-risk cows for preventive intervention and informing preventive nutritional strategies.
	Preventive treatment against ketosis.	Intervention for cows at risk for ketosis based on effective molecules like monensin.	The expert panel identified a significant gap in current management, noting an unmet need for effective, cow-level preventive interventions (expert panel consensus).	Complements the preventive program by adding targeted intervention for at-risk animals to the overall herd approach.
	Data analysis software.	Track and analyze key metrics such as milk yield, ketone levels, and BCS to identify trends and optimize management strategies.	The data integration and analysis were highlighted as fundamental (expert panel consensus); the data-driven decision-making led to effective ketosis intervention [22].	Essential for identifying trends, at-risk cows, and evaluating the effectiveness of preventive interventions.
**Communication**	Veterinarian–farmer collaboration.	Clear communication and shared decision-making between veterinarians and farmers for effective implementation of preventive strategies.	Effective communication and collaboration are essential (expert panel consensus) and [16,18]. Motivational interviewing is a key capability of veterinarians [33,34].	Enables the successful implementation and monitoring of preventive strategies. Evidence-based preventive solutions are only effective when they are properly implemented through clear communication and collaboration.

**Table 2:** Comprehensive ketosis management toolbox primarily for the farmer: integrating expert recommendations and preventive strategies.

Tool Type	Specific Tool/Approach	Description	Expert Panel Emphasis/Literature Support	Preventive Intervention Potential
**Diagnostic**	Milk ketone measuring and fat-to-protein ratio.	Rapid on-farm assessment of milk BHBA levels. Milk BHB offers high diagnostic utility with reported sensitivity and specificity, often exceeding 80% and 90%, respectively, when compared to blood BHB. Testing needs to be standardized per herd, and preferably in the first week post-calving. The fat-to-protein ratio measured in line with the milking process helps to identify cows with metabolic issues in early lactation.	[10,35,45].	Limited direct preventive potential, primarily for diagnosis.
	Behavior analysis.	Recommendation to validate sensor tools for reliable detection of ketosis.	Expert panel consensus.	Exhibits restricted direct prophylaxis; however, it offers a valuable mechanism for the early identification of SCK in animals where the condition might otherwise remain undiagnosed.
**Monitoring**	In milk testing for ketosis and fat-to-protein ratio screening.	Herd-level screening for subclinical ketosis can be incorporated into routine milk recording or even in in-line milk recording. Routine prevalence testing via milk in all or in batches of freshly calved cows.	In-line milk tests, cow-side biomarker assessments, automated monitoring systems, and real-time data analysis advocate for early detection and estimation of ketosis prevalence (expert panel consensus) and [22,45].	Supports prevention through monitoring.
	Monitoring of fresh cow health.	Close observation by clinical examination, by inviting the vet, by utilizing farm staff, or by health alert systems in the first weeks of lactation for early detection of ketosis.	Early detection is critical (expert panel), and [39] the validation of health monitoring systems is crucial (expert panel consensus).	Supports early intervention to minimize the impact of ketosis.
	Monitoring of risk factors in dry cows using algorithms and artificial intelligence.	Close observation of cows before the onset of negative energy balance.	The routine detection of ketosis is critical (expert panel consensus).	Supports preventive preintervention to minimize the prevalence of ketosis.
**Treatment/execution**	Propylene glycol oral glucose precursors.	Targeted oral propylene glycol treatment in subclinical cases has been shown to reduce the risk of displaced abomasum by 40% and increase milk yield by 0.5 kg/day during the first 30 days of lactation compared to untreated.	[40,41,42].	Mainly used as treatment of ketosis for 3–5 days.
	B vitamins (especially B12 or combined with butaphosphan).	Injections to support metabolic function.	[41,42,44].	Supportive therapy may contribute to prevention if used strategically alongside other measures.
**Management/** **Prevention**	Body condition scoring (BCS).	Regular monitoring of BCS throughout lactation helps identify cows at risk and guide nutritional adjustments.	Expert panel consensus.	Important for identifying at-risk cows for preventive intervention and informing preventive nutritional strategies.
	Prevention of excessive energy intake in the dry cow period.	Close calculation of the energy intake of the dry cow ration.	Ref. [4] and expert panel consensus.	This prevents increased fat storage, which causes a high risk of ketosis.
	Active transition cow management (strategy)/program present on the farm.	A comprehensive approach that encompasses nutrition, housing, and monitoring during the critical transition period.	Meticulous dietary management during the close-up dry period is a key aspect of prevention (expert panel consensus); proactive health management is important [24].	Important for minimizing the risks of NEB and ketosis during the transition period at the herd level.
	Implementation of positive welfare elements in the transition period.	Complementary preventive strategies with positive welfare interventions.	[47,48].	Enhancing the resilience of animals by creating space for behavior preferences and (heat) stress reduction.
	Preventive treatment against ketosis.	Intervention for cows at risk for ketosis based on effective molecules like monensin.	There is an unmet need for effective preventive intervention at the cow level (expert panel consensus).	Complements the preventive program by adding targeted intervention for at-risk animals to the overall herd approach.
	Nutritional management during the fresh cow period.	An adequate physical form of the diet should focus on stimulating ruminal activity and chewing behavior in the post-partum period to avoid ruminal acidosis and a concurrent decrease in feed intake.	[49].	Prevention of increased NEB post calving.
	Genetic selection for improved metabolic resilience.	Breeding for cows with greater resistance to metabolic challenges can contribute to long-term herd health.	Expert panel consensus.	Long-term strategy for improving herd-level resistance to ketosis.

## Data Availability

No new data were created or analyzed in this study. Data sharing is not applicable to this article.

## Supplementary Materials

The following supporting information can be downloaded at https://www.mdpi.com/article/10.3390/vetsci13040344/s1.
