# Supplementary material for: The Future Toolbox for Managing Ketosis in Dairy Cow Herds: A European Key Opinion Leader Consensus
Source: Vet Sci. 2026 Apr 1;13(4):344. doi: 10.3390/vetsci13040344 (PMC13119629; doi:10.3390/vetsci13040344)
Supplement: Supplementary file 1 [file vetsci-13-00344-s001.zip › vetsci-4177288-supplementary.pdf]

This recommendation translates into a 3-point action plan for veterinarians:

1. Make metabolic health a routine priority:

Address ketosis and overall metabolic health at every herd health visit, ensuring these topics are a standard part of your routine work on every farm. Ensure each farm under your care has a standardized ketosis monitoring protocol and a routine dry cow risk assessment. Use currently available tools and stay up to date by seeking training on new management and monitoring options as they become available.

2. Interpret and communicate results clearly:

Be prepared to interpret results from multiple monitoring methods and farm-specific data. Provide farmers with clear, straightforward summaries of their herd's metabolic health status, including well-defined thresholds for ketosis (validated against 1.2 mmol /L blood BHB) and high-risk cows (body conditions score > 3.5; parity ≥3; problematic previous transition) [7]. Supply monitoring protocols with easy-to-follow decision trees. Make sure that farmers and their staff understand ketosis, know how to interpret monitoring outcomes, and are prepared to carry out and adapt the agreed management plans.

3. Develop communication and coaching skills:

Continuously improve your data analysis and communication skills. Build expertise in motivational interviewing and other effective coaching methods, enabling you to educate and support farmers and their teams in making lasting improvements to herd health.

This recommendation translates into a clear 3-point action plan for farmers as well:

1. Actively monitor for ketosis and metabolic health:

Understand that most cases of ketosis are subclinical and require testing to detect. Commit to regularly monitoring your herd's metabolic health using simple milk or blood ketone tests or validated sensor technologies, as agreed with your veterinarian.

2. Keep accurate records and build team awareness:

Maintain complete, standardized records on your transition cows, and make sure all staff are trained to recognize high-risk animals and effectively carry out monitoring and treatments. Good record-keeping and awareness are the foundation for early detection and timely action.

3. Collaborate, review, and adapt with your vet:

Use your veterinarian as a proactive partner, not just for treating illness but for improving herd health overall. Regularly review monitoring results with your vet and other advisors, and be ready to adapt your protocols and management practices based on new insights and recommendations to continuously improve your herd's health, productivity, and sustainability.
